# Resection of a malignant paraganglioma located behind the retrohepatic segment of the inferior vena cava

**DOI:** 10.1186/1471-2482-13-49

**Published:** 2013-10-29

**Authors:** Changjun Jia, Xinlu Wang, Chaoliu Dai, Xianmin Bu, Songlin Peng, Feng Xu, Yongqing Xu, Yang Zhao

**Affiliations:** 1 Department of General Surgery, China Medical University, 110004 Shenyang, Liaoning Province, P.R. China; 2 Department of Ultrasound Medicine, Shengjing Hospital, China Medical University, 110004 Shenyang, Liaoning Province, P.R. China

**Keywords:** Paraganglioma, Inferior vena cava, Partial hepatectomy, Malignant

## Abstract

**Background:**

Resection of a retrocaval paraganglioma is technically challenging due to limited tumor accessibility and proximity to the vena cava.

**Case presentation:**

A large, malignant paraganglioma was found behind the retrohepatic segment of the inferior vena cava of a 60-year-old male. During resection of this rare paraganglioma, the left lateral lobe of the liver, a portion of the caudate lobe of the liver, and the gallbladder were also removed. Unfortunately, the patient died six months after surgery due to hepatic metastasis.

**Conclusion:**

This case demonstrates that a partial hepatectomy may be necessary to improve tumor accessibility during resection of a retrocaval paraganglioma, particularly if the tumor is proximal to the vena cava. Furthermore, palliative treatments may help prevent tumor recurrence and metastasis of malignant paragangliomas.

## Background

Paragangliomas, also known as extra-adrenal pheochromocytomas, are rare. Moreover, they are usually benign, catecholamine-secreting tumors that arise from chromaffin cells of the sympathetic ganglia [1]. Although most paragangliomas are located along the para-aortic sympathetic chains in the urinary bladder, thoracic cavity, or head and neck, they have also been found to develop in other regions of the body [2]. Resection of paragangliomas can be difficult, due to the complex anatomy and intensive intraoperative hemodynamic control needed, particularly as retrocaval paragangliomas often develop in proximity of the vena cava [3,4]. Correspondingly, meticulous surgical procedures are required for resection of these tumors. In addition, these surgeries are often associated with a high risk of damage to adjacent organs or blood vessels. Although several reports have described the successful resection of retrocaval paragangliomas [3-6], these tumors were either relatively small (< 5 cm) or were located near renal veins.

Here, a rare malignant paraganglioma located behind the retrohepatic segment of the inferior vena cava (IVC) is described. By performing a partial hepatectomy, the retrocaval tumor was successfully resected without bypass of the vena cava.

## Case presentation

A 60-year-old man presented with a 2-month history of persistent right epigastric pain and a weight loss of 9 kg, without fever, headaches, palpitations, and sweating attacks. His two brothers had previously died of esophageal cancer and gastric cancer. At a local hospital, a computed tomography (CT) scan without using contrast medium revealed a large retroperitoneal mass that was characterized by a non-homogeneous appearance and slightly low density. Magnetic resonance imaging showed both hypo- and hyper-signal intensities on T1-weighted images, whereas only hyper-signal intensity was observed on T2-weighted images. Compression of the hepatic segment of the IVC was also observed. Due to the complex anatomy of the tumor region and the need for resection, the patient was referred to a tertiary unit for further oncological and surgical assessment.

Upon admission, the patient denied any history of hypertension or any other cardiovascular disease. A general physical examination findings were normal. The patient’s blood pressure varied at normal level (110-130/69-86 mmHg). Blood and urine analyses, liver and kidney function, and clotting time, were also normal.

The patient’s fasting blood glucose level was 7.34 mmol/L (reference range: 3.9–6.11 mmol/L) and glycosylated hemoglobin level was 7.2 mg/ dL. Serum levels of tumor markers, including carcinoembryonic antigen, alpha-fe-toprotein, and carbohydrate, were normal. Preoperative levels of adrenocorticotropic hormone were assayed at various time points: 15.98 pg/mL at 0:00, 20.25 pg/mL at 8:00, and 14.40 pg/mL at 16:00. Plasma renin activity was 1.30 ng/mL (0.05–2.86 in the lying position), angiotensin II was 31.50 pg/mL (16.2–64.2, lying down), aldosterone was 91.00 pg/mL (59–174, lying down), and serum levels of cortisol were 7.95 μg/dL at 0:00, 14.53 μg/dL at 8:00, and 6.44 μg/dL at 16:00. Levels of vanillylmandelic acid and urine volume after 24 h were 31.41 mg (1.4–6.5 mg) and 2.05 L, respectively.

**Figure 1:**
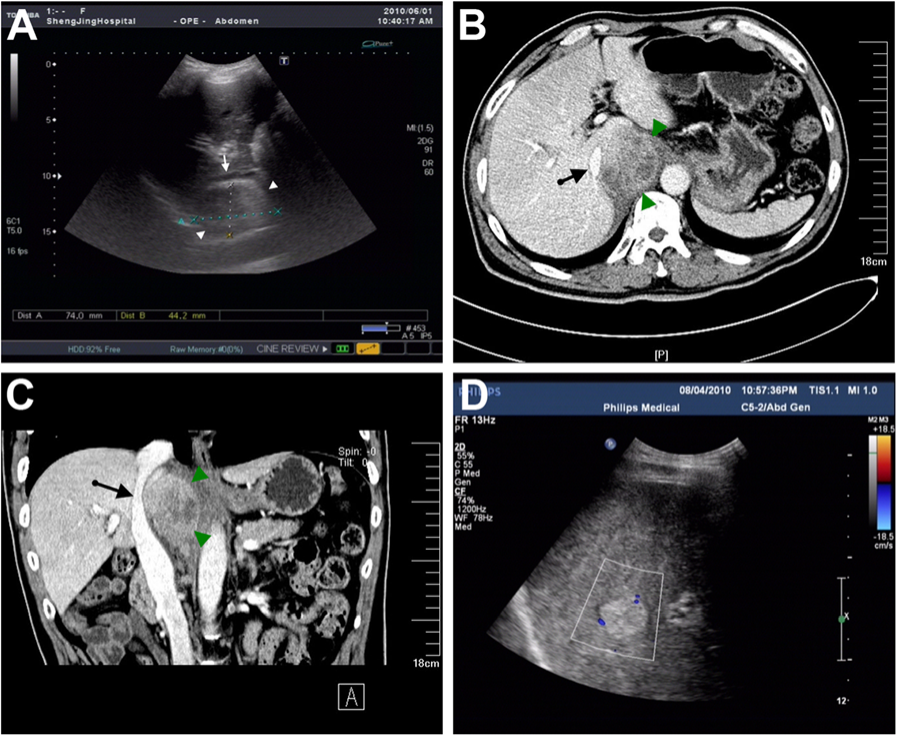
**Diagnostic imaging of the retroperitoneal paraganglioma. (A)** Abdominal ultrasonography showed a retrocaval mass (indicated by arrowheads) present in the right parasagittal sonographic section. The mass compressed the IVC (indicated by an white arrow) in an anterior direction. **(B)** Axial contrast-enhanced CT scan showed a non-homogeneously enhanced retrocaval mass (indicated by arrowheads) with necrotic cysts. **(C)** Coronal contrast-enhanced CT scan showed anterior displacement and extrinsic compression of the IVC (indicated by an black arrow) by the retrocaval mass (indicated by arrowheads). **(D)** Postoperative abdominal US scan showed multifocal hepatic metastatic lesions (shown in blue).

Ultrasonography revealed a 7.4 cm × 4.5 cm well-demarcated solid mass with mixed echoes (a mosaic of medium and low signals) located between the upper abdominal IVC and the aortic artery (Figure 1A). The hepatic segment of the IVC was also compressed and shifted forward. A contrast-enhanced CT with 3D reconstruction scan showed a 6.5 cm × 4.8 cm mass with non-homogeneous density located behind the retrohepatic segment of the IVC (Figure 1B&C). The mass was well-delineated, exhibited non-homogeneous enhancement with multiple necrotic cysts, and the adjacent lymph nodes were not swollen. Compression of the right adrenal gland, caudate lobe, and IVC, and invasion of the right side of the diaphragm horn were also observed. The retrohepatic segment of the IVC was mostly compressed, with the left vessel wall attached to the mass. There was no clear boundary between the left vessel wall and the mass. In addition, there were no filling defects visualized in the vessel lumen, and no distant metastasis was detected. Thus, a preoperative diagnosis of primary retroperitoneal tumor (except for normotensive ectopic pheochromocytoma) was made.

**Figure 2:**
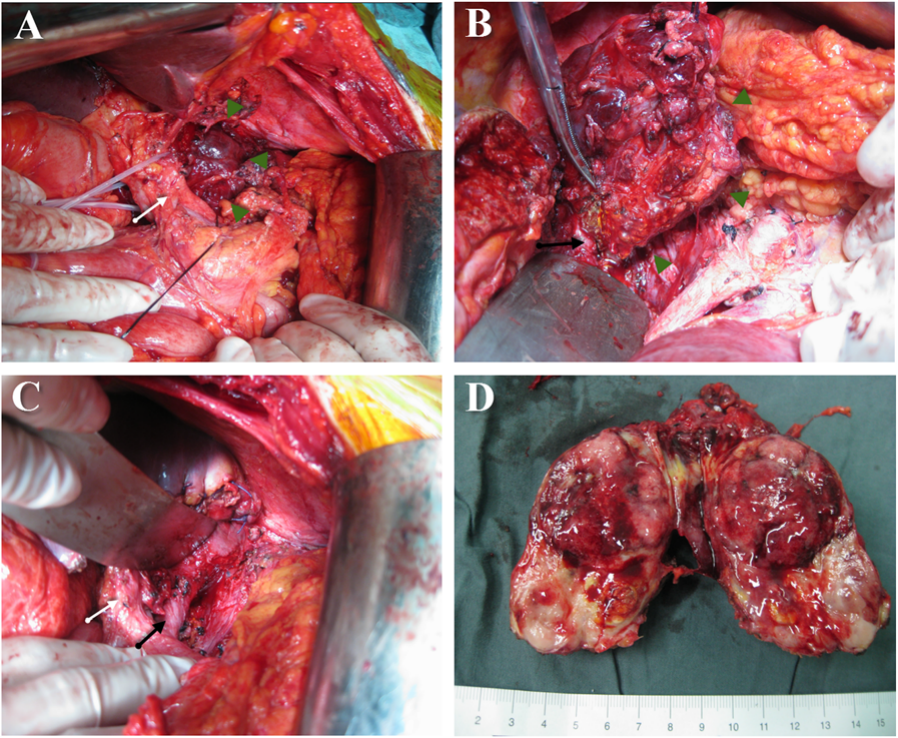
**Intraoperative imaging of the retroperitoneal paraganglioma. (A)** The tumor (indicated by arrowheads) was found behind the retrohepatic segment of the IVC with the hepatic hilum (indicated by an white arrow) lifted in an anterior direction. **(B)** After the gallbladder and the hepatic left lateral lobe were resected, the IVC (indicated by an black arrow) was carefully mobilized clockwise in a cephalad and dorsal direction up to the level of the second hepatic hilum via a left lateral side approach. **(C)** After the tumor was removed, the IVC (indicated by an black arrow) and the prevertebral space become fully visible. **(D)** Specimen of the retroperitoneal paraganglioma.

Prior to undergoing a laparotomy, the patient completed 3-day volume expansion therapy with artificial colloid solution. During surgical exploration, a generous right subcostal incision was made. The mass of interest was found behind the retrohepatic portion of the IVC. The adjacent lymph nodes were not swollen. Compression of the IVC was observed, with the hepatic hilum lifted in an anterior direction (Figure 2A). The tumor and retrohepatic portion of the IVC were fully exposed by resection of the hepatic left lateral lobe. The tumor was identified from the renal level to the second hepatic hilum, and a portion of the caudate lobe was found to be affected. Consequently, the affected portion of the caudate lobe were resected, and extensive dissection of the IVC followed. Briefly, the IVC was carefully mobilized clockwise in a cephalad and dorsal direction up to the level of the second hepatic hilum via a left lateral side approach. Finally, the prevertebral space was divided carefully behind the tumor, and a portion of the affected diaphragm tissue on the right side, as well as the surrounding diaphragmatic muscle, were removed (Figure 2B&C). Thus, the tumor was successfully removed along with the left lateral lobe of the liver, a portion of the caudate lobe of the liver, and the gallbladder.

Intraoperatively, there was two sharp changes in hemodynamic status of the patients observed during manipulation of the tumour (blood pressure rose to 150-160/90-100 mmHg), which was treated with nicardipine hydrochloride and sodium nitroprusside. Esmolol hydrochloride was used to decrease the heart rate. After the tumor was resected, the blood pressure dropped to 70/50 mmHg and norepinephrine was pumped continuously one half hour. Blood pressure rose and steadied at 110/85 mmHg in the end.

**Figure 3:**
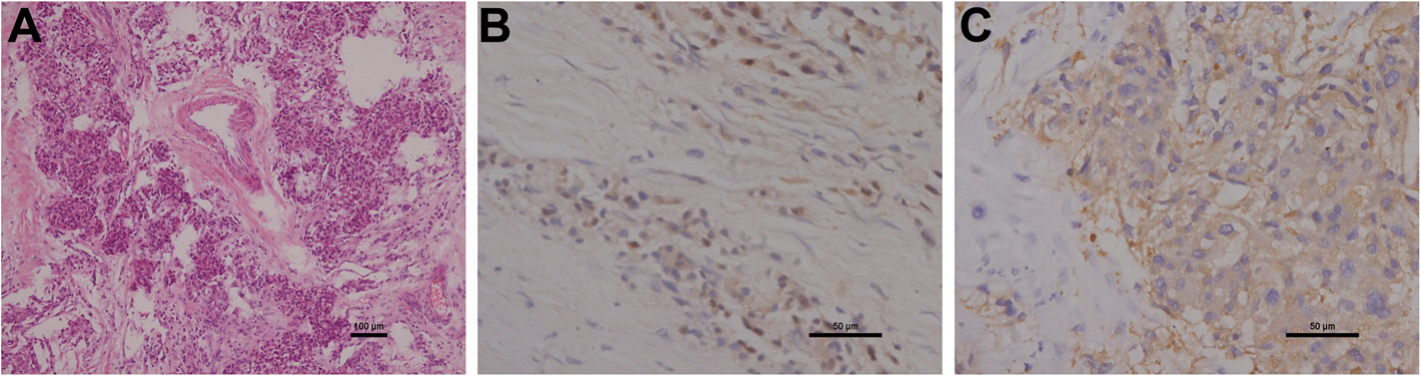
**Histological and immunohistochemical analyses of the retroperitoneal paraganglioma. (A)** Hematoxylin and eosin staining of the resected specimen (magnification, 100×; scale bar = 100 μm). **(B and C)** Immunohistochemical staining for **(B)** S100 protein and **(C)** synaptophysin (magnification, 400×; scale bar = 50 μm).

The resected tumor (8 cm × 6 cm × 4 cm) was solid, nodular, and encapsulated. On the tumor surface, many tortuous vessels and some localized necrotic tissue were observed (Figure 2D). Macroscopically, tumor invasion into the liver and the adrenal gland was not apparent. However, microscopically, the tumor was found to have invaded the caudate lobe. Histological analysis identified alveolar-like structures with many vascular septa within the tumor (Figure 3A). In addition, the tumor cells contained fine eosinophilic granules, while cell mitosis and nuclear atypia were infrequent. Immunohistochemical analyses demonstrated that the tumor cells were negative for α-smooth muscle actin (SMA), and positive for chromogranin A (CgA), synaptophysin (Syn), S-100, cytokeratin (CK), and CD34 (Figure 3B&C).

By 3 days after surgery, the patient undergone closed thoracic drainage for the right side of massive pleural effusion and aerothorax with the obviously compression of the right lung. The closed thoracic drainage tube was removed on postoperative day 18 and the patient was discharged on postoperative day 21. Postoperative adjuvant chemotherapy CVD-protocol (cyclophosphamide 750 mg/m^2^, vincristine 1.4 mg/m^2^, and dacarbazine 600 mg/m^2^ on Day 1 and dacarbaine 600 mg/m^2^ on Day 2; every 21 to 28 days) was recommended; however, the patient refused. During the follow-up period, the patient’s initial symptoms of paraganglioma rapidly disappeared. However, 3 months later, ultrasonography revealed hepatic metastasis (Figure 1D). The patient refused any other treatment and died 3 months later.

## Discussion

Paragangliomas can develop anywhere along the midline of the retroperitoneum. The exact incidence of retroperitoneal paragangliomas is unknown, although males are typically affected more frequently than females. In addition, most patients are diagnosed between 30 and 45 years of age [7]. Clinically, patients with a retroperitoneal paraganglioma often present with back pain or a palpable mass [8]. Approximately 10% of cases have distant metastases detected at the time of diagnosis [9]. Only a subset of paragangliomas is clinically functional and often exhibit signs and symptoms consistent with actively secretion of catecholamine, including headaches, sweating, palpitation, and hypertension [7]. The present case represents a rare normotensive malignant paraganglioma that developed in a relatively aged man, only presented with abdominal pain.

Based on an increase in the level of urine vanillylmandelic acid after 24 h and sharp fluctuation of intraoperative blood pressure, the normotensive paraganlioma is functional in the present case. Prior to the resection of pheochromocytoma or paraganglioma, alpha blocker is still recommended. However, the effect has never been tested in a randomized clinical trial. The necessity of preoperative alpha blocker remains uncertain for the normotensive or asymptomatic pheochromocytom [10]. Shao *et al*. [11] considered that preoperative alpha-adrenoceptor antagonist didn’t have benefit in maintaining intraoperative hemodynamic stability in patients with normotensive pheochromocytoma. It may increase the use of vasoactive drugs and colloid infusion. Groeben *et al*. [12] also reported that there was no correlation between the individual dose of phenoxybenzamine and the maximum blood pressure in 200 consecutive resections of catecholamine-producing tumors. In the present case, alpha-blocker wasn’t used prior to the operation. Although the intraoperative blood pressure varied sharply, there wasn’t malignant hypertension. The operation and anaesthesia process were controllable, which may be related with the improvement of surgical techniques, diagnostic tools and highly effective short acting substances to control hemodynamics intraoperatively [12].

Conventional treatment for paragangliomas typically involves complete surgical excision, while surgical debulking is considered a mainstay of palliative therapy for malignant paragangliomas [13]. In some cases, complete excision is difficult due to the highly vascular nature of paragangliomas and their proximity to major blood vessels. While some authors have reported the successful resection of retrocaval paragangliomas with or without the need for vena cava reconstruction [3-6], the location of the present paraganglioma behind the retrohepatic portion of the IVC made it difficult to resect the tumor completely. A combined partial hepatectomy was performed to observe the relationship between the tumor and IVC directly. If extensive invasion of the IVC has occurred, vena cava repair or reconstruction may be required [5]. Moreover, if the IVC is completely occluded due to compression and invasion by a tumor, the IVC can be ligated after tumor resection [5]. If the tumor is large and extends across the entire region of the retrohepatic IVC, or invades surrounding liver tissue as observed in the present case, then a combined partial hepatectomy may be more suitable. Although both laparoscopic and open resection of retroperitoneal paragangliomas are possible, a retrocaval tumor > 5 cm is not considered a candidate for laparoscopic surgery [3].

Although paragangliomas are usually benign, 30–50% of all retroperitoneal paragangliomas are malignant [14]. No definitive tests are currently available to differentiate between benign and malignant paragangliomas. Malignancy can often only be confirmed by detecting local invasion of surrounding structures upon examination at the time of resection, or by detecting the presence of metastases [1]. Furthermore, although histological and immunohistochemical findings do not permit a definitive diagnosis of malignancy, several factors have been associated with malignancy. These include a tumor weight > 80 g, high concentration of dopamine proximal to the tumor, tumor size > 5 cm, presence of confluent tumor necrosis, and a younger patient age [15]. In the present case, tumor invasion into the caudate lobe of the liver was observed microscopically, and hepatic metastasis was detected in the postoperative follow-up period.

Although *en bloc* resection was performed to remove the affected surrounding hepatic lobe and diaphragm tissue, it did not extend the life of the patient. Had the malignant nature of the tumor been identified, then the pharmacologic control of symptoms, targeted methods, and systemic antineoplastic therapy could have been applied [16]. The most effective chemotherapy regime is the CVD-protocol, as partial - and in few cases complete- response in up to 50-55% of the patients [17]. In contrast, palliative treatments for patients with advanced paragangliomas remain limited [1].

## Conclusions

Here, we report an extremely rare case of malignant paraganglioma, in which the tumor was located behind the retrohepatic segment of the IVC. The paraganglioma was successfully resected, along with the left lateral lobe and caudate lobe of the liver. This case demonstrates that a partial hepatectomy can improve tumor accessibility, particularly if the tumor is proximal to the vena cava. In addition, palliative treatments may help prevent postoperative tumor recurrence and metastasis.

## Consent

Written informed consent was obtained from the patient for publication of this case report and any accompanying images prior to his death. A copy of the written consent is available for review by the Editor of this journal.

## Competing interests

The authors declare that they have no competing interests.

## Authors’ contributions

CJ and XW prepared the manuscript and performed the literature search; CD and XB corrected and revised the manuscript; CD, CJ, YX, and YZ treated and observed the patient; XW provided the radiographic and ultrasound images; and SP and FX performed the histopathological and immunohistochemical examinations. All authors read and approved the final manuscript.

## Pre-publication history

The pre-publication history for this paper can be accessed here:

http://www.biomedcentral.com/1471-2482/13/49/prepub
